# *Drosophila* FGFR/Htl signaling shapes embryonic glia to phagocytose apoptotic neurons

**DOI:** 10.1038/s41420-023-01382-5

**Published:** 2023-03-10

**Authors:** Malak Ayoub, Li-mor David, Boris Shklyar, Ketty Hakim-Mishnaevski, Estee Kurant

**Affiliations:** 1 Department of Human Biology, Faculty of Natural Sciences, University of Haifa, 3498838 Haifa, Israel; 2 Bioimaging Unit, Faculty of Natural Sciences, University of Haifa, 3498838 Haifa, Israel

**Keywords:** Cell death, Embryogenesis

## Abstract

Glial phagocytosis of apoptotic neurons is crucial for development and proper function of the central nervous system. Relying on transmembrane receptors located on their protrusions, phagocytic glia recognize and engulf apoptotic debris. Like vertebrate microglia, *Drosophila* phagocytic glial cells form an elaborate network in the developing brain to reach and remove apoptotic neurons. However, the mechanisms controlling creation of the branched morphology of these glial cells critical for their phagocytic ability remain unknown. Here, we demonstrate that during early embryogenesis, the *Drosophila* fibroblast growth factor receptor (FGFR) Heartless (Htl) and its ligand Pyramus are essential in glial cells for the formation of glial extensions, the presence of which strongly affects glial phagocytosis of apoptotic neurons during later stages of embryonic development. Reduction in Htl pathway activity results in shorter lengths and lower complexity of glial branches, thereby disrupting the glial network. Our work thus illuminates the important role Htl signaling plays in glial subcellular morphogenesis and in establishing glial phagocytic ability.

## Introduction

Massive neuronal apoptosis and subsequent removal of apoptotic neurons in the developing central nervous system (CNS) are critical for functional brain formation in vertebrates and *Drosophila* [1–3]. In vertebrates, phagocytic microglia of mesodermal origin serve as the main cleaners in the developing CNS [4, 5]. These are motile cells, which constantly extend protrusions that probe the environment to accurately and efficiently remove apoptotic neurons [4, 5]. During *Drosophila* embryogenesis, apoptotic neurons are cleared by phagocytic glia of ectodermal origin, which are functionally and molecularly similar to their vertebrate counterparts [6, 7]. Like microglia, *Drosophila* glial cells are branched and form an elaborate network, which constantly probes the environment by sending out cell protrusions [8]. Two main glial transmembrane receptors Draper [9] and SIMU [10], the expression of which is developmentally regulated, mediate distinct steps of phagocytosis of apoptotic neurons in the embryonic CNS. Specifically, SIMU mediates recognition and engulfment, while Draper is involved in phagosome maturation and degradation [10]. Although interactions between these receptors and their ligands exposed on apoptotic debris provide the basis for phagocytosis, the role of glial cell shape in this process has yet to be examined.

Glial differentiation is initiated by a key regulator of glial cell fate, the transcription factor Glial Cells Missing (GCM) and its homolog GCM2, which are transiently expressed in precursors of macrophages and glia during early embryogenesis [11–13]. At stage 10 of embryogenesis, GCM/GCM2 induce expression of Reversed Polarity (REPO), another transcription factor that is required for the differentiation of all types of lateral (not midline) glia [14, 15]. While these crucial regulators of glial development activate multiple genes, it remains unclear which of them specifically control formation of the unique branched shape of phagocytic glia.

The *heartless (htl)* gene encodes a *Drosophila* homolog of Fibroblast Growth Factor (FGF) receptor (FGFR), which was shown to be involved in cell migration and tissue morphogenesis during embryogenesis including glial enwrapping of axonal tracks [16–20]. In addition, the role of Htl was established in post-embryonic gliogenesis, and shown to mostly affect the proliferation and migration of glial cells [21–23]. *pyramus (pyr) and thisbe (ths)* encode two ligands of Htl [18] that share homology with the FGF8 family of vertebrate FGFs [24]. In the eye disc, Pyr and Ths coordinate the Htl signaling involved in glial proliferation, migration and differentiation [21]. During larval stages, the Htl pathway was reported to regulate astrocytic glia size and their growth into the synapse-rich neuropil [25]. At the same time, the role of Htl signaling at the onset of embryonic glia has yet to be recognized.

Here, we reveal that during early embryogenesis, before neuronal apoptosis begins and subtypes of glia are formed [26], Htl signaling is autonomously required for the subcellular morphogenesis of glial cells, an event that, in turn, influences glial phagocytosis of apoptotic neurons at later embryonic stages. We demonstrate that Pyr released from glial cells is the main ligand required for Htl pathway activation. Strikingly, cell shape analysis of embryonic glia revealed that the Htl pathway regulates the length and complexity of glial cell processes. We further show that this step is critical for the formation of a functional glial network and for establishment of glial phagocytic ability, both of which are involved in the clearance of apoptotic neurons during development. These findings emphasize the importance of subcellular morphogenesis for the proper function of phagocytes in apoptotic cell clearance.

## Results

### *pyr* is required for glial phagocytosis of apoptotic neurons in the embryonic CNS

**Fig. 1:**
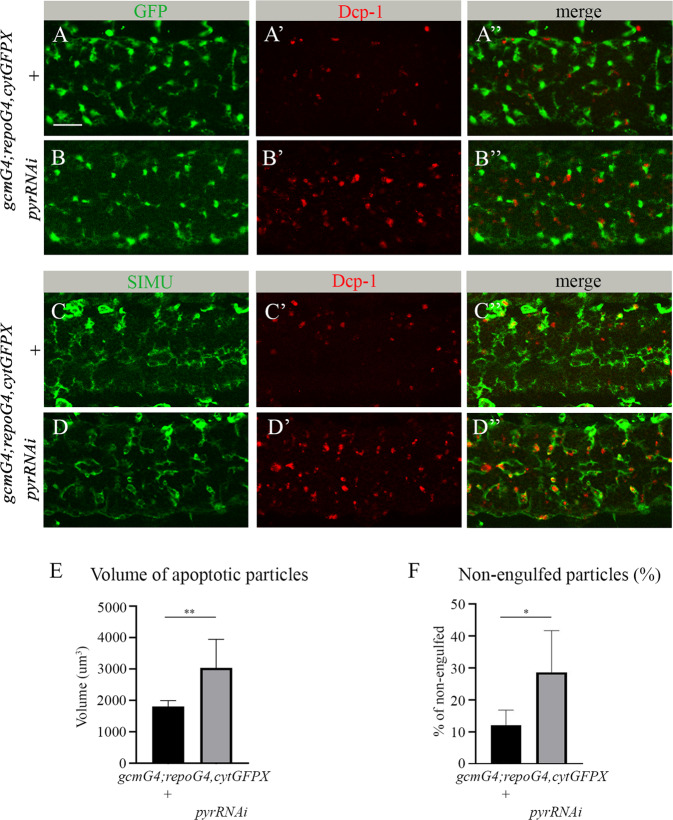
*pyr* RNAi knock down impairs clearance of apoptotic cells by embryonic glia. **A**-**D**” Single panes of the embryonic CNS at stage 16; ventral view. Bar, 20 µm. **A**-**A**”, **C**-**C**” Control embryos (*gcmGal4/+;repoGal4,cytGFP/+*) and **B**-**B**”, **D**-**D**” embryos expressing *pyr* RNAi in glia (*gcmGal4/+;repoGal4,cytGFP/UASpyrRNAi*) stained with anti-GFP (**A**, **A**”, **B**, **B**”, green) or anti-SIMU (**C**, **C**”, **D**, **D**”, green) and anti-Dcp-1 (red) antibodies. **E** Mean total volume of apoptotic particles within CNS sections and **F** percentages of apoptotic cell volume not engulfed by glia ± SEM, *n* = 9 (control), *n* = 8 (*pyr* RNAi). Statistical significance was analyzed employing Student’s *t* test, **p < 0.01, *p < 0.05. Note the significant increase in volume of apoptotic cells and higher percentage of non-engulfed particles in embryos expressing *pyr*RNAi in glia.

To discover new genes involved in glial phagocytosis of apoptotic neurons during *Drosophila* embryogenesis, we took a candidate gene approach. We focused on ten candidates that are expressed in embryonic glia (based on Flybase expression data; Table 1). To test whether a given gene is involved in glial phagocytosis of apoptotic neurons without interfering with its possible function outside the CNS, we specifically knocked down the expression of that gene in embryonic glia using an inducible RNAi construct simultaneously driven with the *gcmGal4* and *repoGal4* drivers. Since *repo* is continuously expressed in glia following the early and transient expression of *gcm* [27, 28], we used these drivers to knock down expression of candidate genes at all embryonic stages of glial development. To evaluate glial phagocytosis, apoptotic cells were labeled with anti-activated caspase 3 antibodies (anti-Dcp-1), while glial cells were visualized with cytoplasmic GFP (Fig. 1A-B”). Using Imaris software, we measured the volumes of apoptotic cells in the designated area (Figs. 1E and S1). Our data revealed that, when compared to other candidates, glia-specific RNAi knock down of *pyr* resulted in the most significant increase in apoptotic cell volume, as compared to the wild type (Figs. 1A-B”, E and S1).

This observation prompted us to further investigate the role of Pyr and the Htl pathway in glial phagocytosis of apoptotic neurons. To determine whether or not apoptotic cells were engulfed by glial cells, we used antibodies that recognize the transmembrane receptor SIMU, a marker of glial cell membranes (Fig. 1C, C”, D, D”). Such labeling allowed us to approximate the percentage of non-engulfed particles (Figs. 1F and S2, Movies 1 and 2). In control embryos, most of the apoptotic debris appeared to be engulfed by glial cells (Fig. 1C-C”, F), whereas in *pyr* RNAi knock down embryos, many of the apoptotic particles were found outside glial cells (Fig. 1D-D”, F), implying a failure in recognition and/or engulfment by glial cells. Finally, while Pyr is a secreted protein and can originate in different cells, given how we knocked down its expression specifically in glial cells, we could conclude that it functions in auto- or paracrine manner.

### Htl is essential for clearance of apoptotic neurons by embryonic glia

**Fig. 2:**
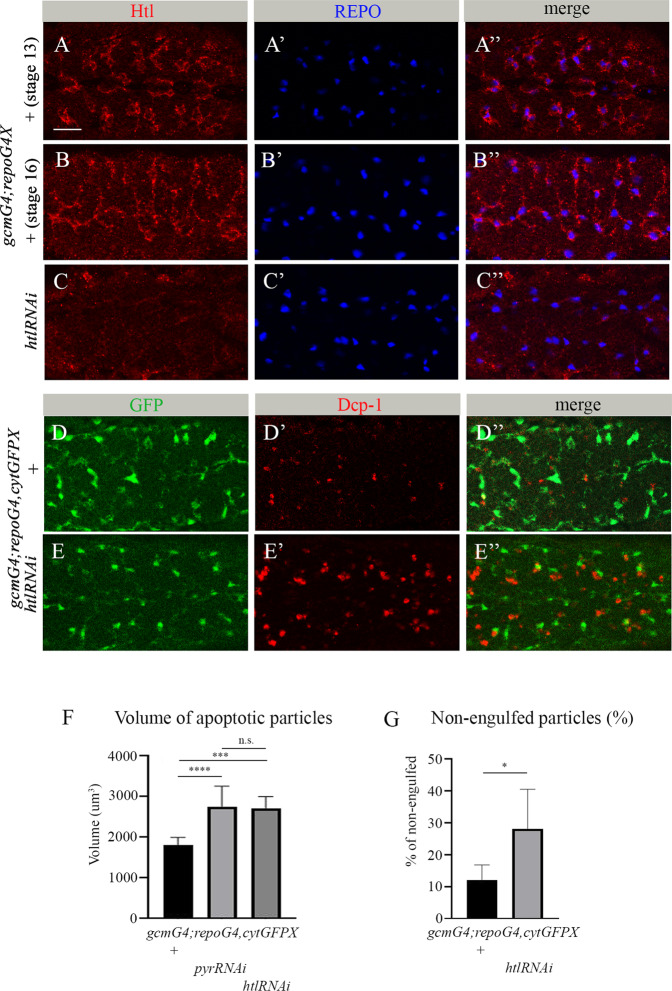
*htl* is required in embryonic glia for phagocytosis of apoptotic neurons. **A**-**E**” Single panes of the embryonic CNS; ventral view. Bar, 20 µm. **A**-**B**” Control embryos at stage 13 (**A**-**A**”) and stage 16 (**B**-**B**”) and (**C**-**C**”) embryos at stage 16 expressing *htl* RNAi in glia (*gcmGal4/+;repoGal4,cytGFP/UAShtlRNAi*) stained with anti-Htl (red) and anti-REPO (blue) antibodies. **D**-**D**” Stage 16 control embryos and **E**-**E**” embryos expressing *htl* RNAi in glia (*gcmGal4/+;repoGal4,cytGFP/UAShtlRNAi*) stained with anti-GFP (green) and anti-Dcp-1 (red) antibodies. **F** Mean total volume of apoptotic particles within stacks of the CNS and **G** percentages of apoptotic cell volume that is not engulfed by glia ± SEM, *n* = 9 (control), *n* = 6 (*htl* RNAi). Statistical significance was analyzed employing one-way ANOVA (**F**) and Student’s *t* test (**G**), ****p < 0.0001, ***p < 0.001, *p < 0.05, n.s. (non-significant) *p* > 0.05. Note the significant increase in volume of apoptotic cells and higher percentage of non-engulfed particles in embryos expressing *htl* RNAi in glia.

Pyr is a known Htl ligand. Therefore, we tested the role of Htl in glial phagocytosis of apoptotic neurons. Htl expression begins in embryonic mesoderm at very early stages and is required for normal embryogenesis [20, 29]. *htl* mutant embryos exhibit multiple defects in the development of different systems and organs, and do not survive to larval stages. To determine whether Htl is involved in glial phagocytosis, we first examined its expression in embryonic glia using anti-Htl antibodies. We detected strong staining of the Htl protein on the membranes of glial cells labeled with nuclear anti-REPO antibodies (Fig. 2A-B”). To knock down *htl* specifically in glia, an *htl* RNAi construct was induced with the *gcmGal4* and *repoGal4* drivers simultaneously; the reduction in Htl levels was confirmed with anti-Htl antibodies (Fig. 2C, C”). Embryos expressing lower levels of Htl exhibited significant increases in the volume of apoptotic particles in the CNS (Fig. 2E-E”, F), as compared to control embryos (Fig. 2D-D”, F). Similar findings were seen upon glia-specific RNAi knock down of *pyr*. Also similar to *pyr* knock down, *htl* RNAi embryos exhibited a large number of apoptotic particles outside glial cells (Fig. 2E”, G), suggesting that Htl is required for recognition and/or engulfment of apoptotic neurons. These results suggest that Htl and Pyr function in glia in the same pathway to regulate apoptotic cell clearance.

### Reduced Htl signaling does not affect embryonic neuron and glia survival

**Fig. 3:**
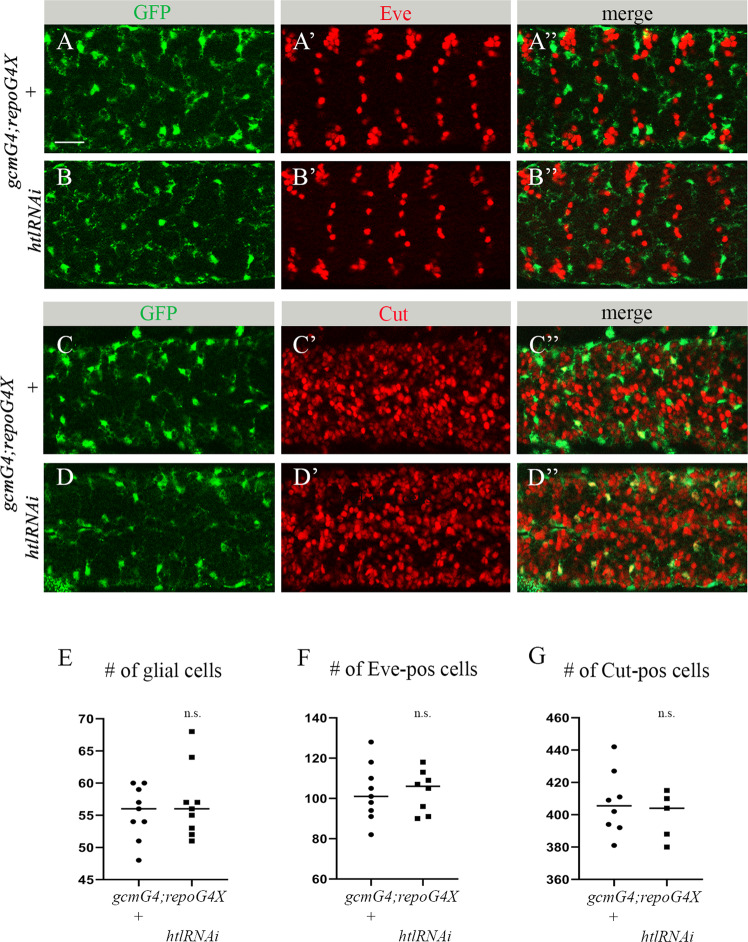
Decreased *htl* in embryonic glia does not affect survival of embryonic neurons and glia. **A**-**D**” Single panes of the embryonic CNS at stage 16; ventral view. Bar, 20 µm. **A**-**A**”, **C**-**C**” Control embryos (*gcmGal4/+;repoGal4,cytGFP/+*) and **B**-**B**”, **D**-**D**” embryos carrying glia-specific *htl* RNAi (*gcmGal4/+;repoGal4,cytGFP/UAShtlRNAi*) stained with anti-GFP (green) and anti-Eve (**A**’, **A**”, **B**’, **B**”, red) or anti-Cut (**C**’, **C**”, **D**’, **D**”, red) antibodies. Quantification of (**E**) REPO-positive glial cells, (**F**) Eve-positive neurons and (**G**) Cut-positive neurons in control and *htl* RNAi knock down embryos. Graphs represent mean total number of REPO-, Eve- or Cut-positive cells within stacks of the CNS ± SEM, *n* = 9 (control), *n* = 9, 8 and 5 (*htl* RNAi respectively). Statistical significance was analyzed employing Student’s *t* test, n.s. (non-significant) *p* > 0.05. Note there is no difference in neuronal and glial numbers between control and *htl* knock down embryos.

Increased volume of apoptotic debris in vivo may result from the increased cell death of glia and/or neurons or from their impaired phagocytic removal. To distinguish between these possibilities, we first evaluated whether glial cells experienced increased cell death. For this, we counted glial nuclei labeled with anti-REPO antibodies in a designated area of the CNS of *htl* RNAi knock down and control embryos (Fig. 2B’, C’). No significant difference in the number of glial nuclei was detected between mutant and control embryos (Fig. 3E), indicating that the observed increase in apoptotic cell volume was not due to glial cell death. Moreover, using specific markers, Even-skipped (Eve) and CUT, we labeled two different types of embryonic neurons, in control and *htl* RNAi embryos (Fig. 3A-D”). We then counted labeled neurons in the same designated area of the CNS as in the glial nuclei count described above (Fig. 2A’). In both experiments, we did not find any significant differences between *htl* knock down and control embryos (Fig. 3F, G), demonstrating that the increased volume of apoptotic particles in the *htl* RNAi knock down embryos did not result from elevated neuronal cell death. Based on these results, we concluded that the reduction in Htl signaling did not increase cell death in the embryonic CNS but instead interfered with the clearance of the already dead cells.

### *pyr*, *ths and htl* mutants display phagocytosis defects similar to that seen upon *pyr* and *htl* glia- specific RNAi knock down

In addition to the glia-specific RNAi-induced knock down of *htl* and *pyr*, we also examined glial phagocytosis of apoptotic neurons in *htl*, *pyr* and *ths* mutants. In the null *pyr*^*s0439*^ allele, the CAG codon encoding a glutamine residue at position 117 within the FGF domain is converted into a premature stop codon [30]. The *ths*^*759*^ allele contains a deletion of exon 3, intron 3 and part of exon 4 of the *ths* gene, resulting in the absence of an 84 amino acid-long stretch of the FGF core domain [31]. In the *htl*^*zz81*^ allele, there is a deletion of 186 nucleotides, precisely mapped to nucleotides 1901–2087 [32].

**Fig. 4:**
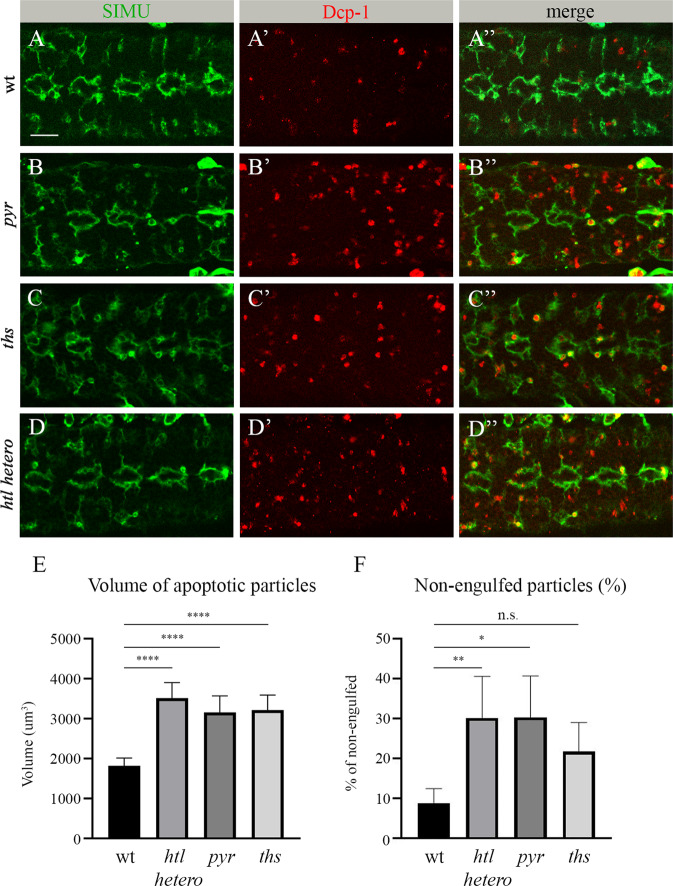
*pyr*, *ths and htl* mutants exhibit impaired glial phagocytosis of apoptotic neurons. **A**-**D**” Single panes of the embryonic CNS at stage 16; ventral view. Bar, 20 µm. **A**-**A**” Control, **B**-**B**” *pyr*^*s0439*^ mutant, **C**-**C**” *ths*^*759*^ mutant and **D**-**D**” *htl*^*zz81*^ heterozygous embryos stained with anti-SIMU (green) and anti-Dcp-1 (red) antibodies. **E** Mean total volume of apoptotic particles within stacks of the CNS and **F** percentages of apoptotic cell volume that is not engulfed by glia ± SEM, *n* = 9. Statistical significance was analyzed employing one-way ANOVA, ****p < 0.0001, **p < 0.01, *p < 0.05, n.s. (non-significant) *p* > 0.05.

All three *pyr*^*s0439*^, *ths*^*759*^ *and htl*^*zz81*^ mutants are embryonic lethal. Moreover, *htl* mutant embryos exhibit abnormal development of the gut and, therefore, it is difficult to phenotypically determine their ages. To examine glial phagocytosis of apoptotic neurons in these mutants, we labeled embryonic glia with anti-SIMU antibodies and apoptotic cells with anti-Dcp-1 antibodies, respectively (Fig. 4A-D”). Homozygous *pyr*^*s0439*^ and *ths*^*759*^ embryos exhibited significantly higher volumes of apoptotic cells, as compared to the wild type (Fig. 4E). *htl*^*zz81*^ homozygous mutant embryos did not reach embryonic stage 16, likely due to multiple developmental defects. As such, we tested the phagocytosis phenotype in heterozygous *htl*^*zz81*^ embryos (Fig. 4D-D”). We found a significant increase in apoptotic particle volume, as compared to the wild type in the *htl* heterozygous background (Fig. 4E). Moreover, in all three mutants, many apoptotic particles were found to be non-engulfed, outside the glial cells (Fig. 4F), strengthening the claim that the genes under consideration, namely *pyr*, *ths and htl*, are involved in the recognition and/or engulfment of apoptotic cells.

### Glial expression of *htl* and *pyr* is required during early embryogenesis to facilitate phagocytosis of apoptotic neurons at late embryonic stages

**Fig. 5:**
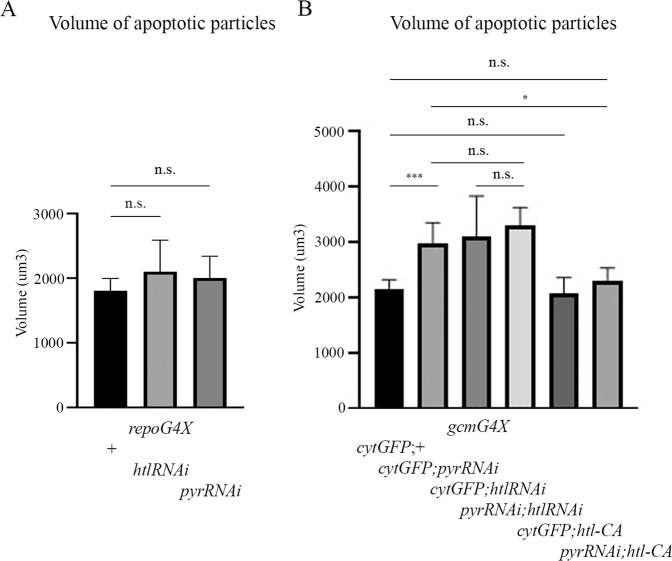
Htl signaling is required at early stages of embryogenesis to control the phagocytic ability of glia. Volume of apoptotic particles within CNS sections of (**A**) control embryos and embryos expressing *htl* RNAi or *pyr* RNAi with *repoGal4* driver; **B** control embryos and embryos expressing *htl* RNAi or *pyr* RNAi or *htl-CA* alone or *htl-CA* with *pyr* RNAi with *gcmGal4* driver. Graphs represent mean total volume of apoptotic particles within stacks of the CNS ± SEM, *n* = 5–9. Statistical significance was analyzed employing one-way ANOVA, ***p < 0.001, *p < 0.05, n.s. (not significant) *p* > 0.05. Note the non-significant increase in the volume of apoptotic cells in *repoGal4-*expressing embryos and the rescue of the *pyr* RNAi phagocytosis phenotype upon *htl-CA* expression at early stages (*gcmGal4/UASpyrRNAi;UAShtl-CA*).

To understand how Htl signaling affects glial phagocytosis of apoptotic cells, we first examined when its function was required during embryogenesis. We knocked down *htl* or *pyr* expressing RNAi constructs specifically at early stages of glial development using the *gcmGal4* driver alone, and the only *repoGal4* driver to knock down gene expression at later stages. *gcmGal4* is expressed from stage 9 up to stage 13 [33], whereas *repoGal4* expression begins at stage 11 and continues into adulthood [14]. Surprisingly, when *pyr* or *htl* RNAi was induced in glia using the *repoGal4* driver, no significant difference in the volume of apoptotic particles was detected in the embryonic CNS between knock down and control embryos (Fig. 5A). However, when we examined those embryos expressing *pyr* or *htl* RNAi with the *gcmGal4* driver only, glial phagocytosis was significantly impaired, as compared to control embryos (Fig. 5B) and to the same extent as upon expression of the same RNAi using both the *gcmGal4* and *repoGal4* drivers (Figs. 1E and 2F). These intriguing results demonstrate that *htl* and *pyr* are required during the early stages of glia formation, even before glial subtypes are specified. This early requirement is then used for the establishment of glial ability to recognize and engulf apoptotic neurons at later stages. In addition, we tested the double knock down of *htl* and *pyr* in glial cells induced by the *gcmGal4* driver and found no additive effect on apoptotic cell clearance, as compared to the single knock down variants (Figs. 5B and 2F). In these experiments, we included an additional UAS construct (*UAScytGFP*) in each single knock down to preserve the same ratio of Gal4 and UAS elements. These results thus demonstrate that Htl and Pyr act in the same pathway.

### Constitutively active Htl signaling rescues defects in glial phagocytosis caused by the lack of *pyr*

To further explore the role of Htl signaling in glial phagocytosis, we expressed a ligand-independent constitutively active form of Htl (Htl-CA) specifically in glia at early stages of embryogenesis using the *gcmGal4* driver. We found no significant difference between volume of apoptotic cells in the CNS of control and Htl over-expressing embryos (Fig. 5B), suggesting that increased Htl signaling does not affect glial phagocytosis of apoptotic neurons. Importantly, however, constitutively active Htl induced with *gcmGal4* at early stages of embryogenesis significantly reduced the volume of apoptotic particles in *pyr* knock down embryos (Fig. 5B), thus demonstrating rescue of the *pyr* phenotype by constitutively active Htl receptor in glia. These results suggest that Pyr is the ligand of the Htl receptor, which is required for activation of Htl signaling in embryonic phagocytic glia. Since *pyr* and *htl* expression was specifically reduced in glia through RNAi-mediated knock down, we can conclude that Htl signaling is required in glial cells at the beginning of glial differentiation for phagocytosis of apoptotic neurons at later stages of embryogenesis.

### Htl signaling is required for the formation of extensions in phagocytic glial cells

**Fig. 6:**
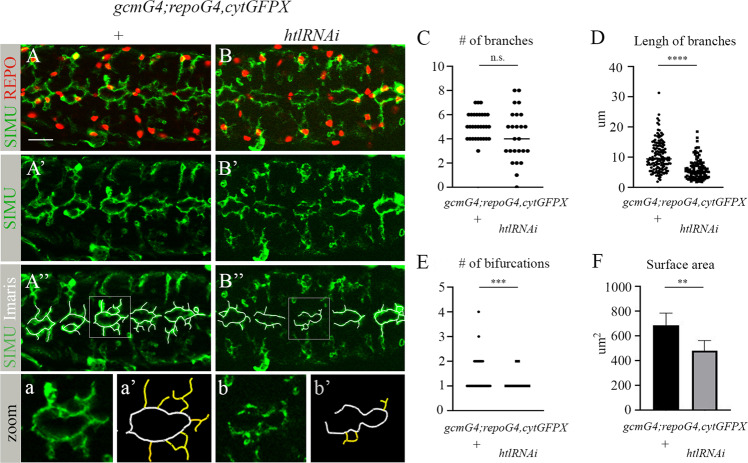
Htl signaling at early embryogenesis is required for formation of the branched shape of phagocytic glia. **A**-**B**” Single panes of the embryonic CNS at stage 16; ventral view. Bar, 20 µm. **A**-**A**”, a, a’ Control embryos (*gcmGal4/UAScytGFP; +/+*) and **B**-**B**”, b, b’ embryos expressing *htlRNAi* with *gcmGal4* (*gcmGal4/UAScytGFP; +/UAShtlRNAi*) stained with anti-SIMU (green) and anti-Repo (red) antibodies. **A**”, **B**” Specific glial branches included in our analysis depicted with white lines using Imaris software. a, a’, b, b’ Zoom-in of rectangular areas of **A**” and **B**”, respectively. Yellow lines mark extensions protruding from the midline, evaluated and presented in graphs. Graphs represent the mean (**C**) number of branches; **D** length of branches; **E** number of bifurcations; and **F** surface area of all yellow-labeled branches ± SEM, *n* = 5. Statistical significance was analyzed employing one-way ANOVA, ****p < 0.0001, ***p < 0.001, **p < 0.01, n.s. (not significant) *p* > 0.05.

To uncover how Htl signaling affects glial phagocytic capacity, we carefully examined the morphology of *htl*-deficient glial cells in the embryonic CNS. As we showed above, there was no reduction in glial cell number, although in the mutant embryos, glial cell protrusions labeled with anti-SIMU antibodies (Fig. 6B) appeared shorter in length and less complex, as compared to control embryo protrusions (Fig. 6A). To quantify this phenotype, we focused on five abdominal segments in each embryo and counted the number of protrusions in glial cells (glial nuclei were marked with anti-REPO antibodies and glial membranes were labeled with anti-SIMU antibodies) (Fig. 6A, B). In our analysis, we only included protrusions protruding from the midline (depicted in Fig. 6a’, b’ in yellow) and not those situated in the midline direction, which appear to be touching/binding neighboring glial cells (depicted in Fig. 6a’, b’ in white). When we counted all outgoing protrusions (i.e., the yellow-labeled protrusions), approximately the same numbers were seen in the *htl* knock down and the control embryos (Fig. 6C), although appeared to be more variable per cell in the knock down embryos than in the controls, where the numbers were between 4 and 7. Furthermore, we measured the mean length of the yellow branches and found them to be significantly shorter in the knock down than in the control embryos (Fig. 6D).

In addition, we determined the percentage of complex branches, specifically, those which start as one protrusion and then bifurcate (Fig. 6E). A significantly lower number of complex branches was found in *htl*-deficient embryos, as compared to control (Fig. 6E). These data indicate that the extensions that form the glial network fail to develop properly in embryos with reduced glial Htl signaling, which likely affects the capacity of glial cells to engulf apoptotic neurons.

**Fig. 7:**
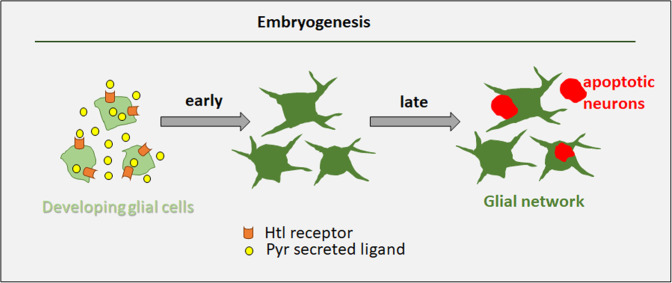
Schematic representation of Htl-regulated formation of the glial network during early embryogenesis. When glial differentiation begins, the Htl receptor is expressed in glial cells. The Htl receptor ligand Pyr is secreted from glial cells and binds Htl on glial membranes, resulting in signaling which is necessary to instruct the cytoskeleton of glial cells to grow and develop extensions. These branched glial cells form an elaborate network that is essential for clearance of apoptotic neurons later in development, when massive neuronal apoptosis takes place in the embryonic CNS. Reduction in Pyr or Htl levels disrupts the glial network and impairs the removal of dying neurons. Accumulation of uncleared dying cells may lead to developmental defects, inflammation and neurodegeneration later on.

To evaluate the surface area of the protrusions, we used Imaris software to measure SIMU-labeled glial surfaces (Fig. S3) and found that the mean surface area was significantly lower in *htl* knock down than in control embryos (Fig. 6F). Thus, Htl signaling is required in developing glia for the initial formation of cell branches necessary for sensing/recognition/uptake of apoptotic debris later in development (Fig. 7). This suggests that the Htl pathway affects glial phagocytosis of apoptotic neurons through the glia-autonomous regulation of subcellular morphogenesis.

## Discussion

The multi-branched shape of different cells, and specifically of cells performing phagocytosis, such as macrophages and microglia, is crucial for their function. Most phagocytic cells present protrusions, which help them approach and engulf targets to be eliminated. However, how this sophisticated cell shape is generated, and how it affects cell phagocytic ability remain unknown. Intuitively, one would think that phagocytic cells generate their protrusions upon sensing target material and as a reaction to the presence of a target. However, we revealed here that the branched shape of *Drosophila* phagocytic glia is defined at very early stages of glial development, long before apoptotic targets appear in the developing CNS. Moreover, this early subcellular morphogenesis determines glial phagocytic capacity required later, when massive neuronal apoptosis takes place in the embryonic CNS (Fig. 7).

To discover new factors involved in glial phagocytosis of apoptotic neurons, we performed a limited candidate screen and found that the Htl pathway plays an important role during early development of phagocytic glia. We found that glia-specific inhibition of Htl signaling causes accumulation of non-engulfed apoptotic neurons in the embryonic CNS. Importantly, we did not find an increase in neuronal and/or glial cell death in response to reduced Htl signaling, indicating that the Htl pathway in glia does not affect cell survival. Rather, compromised Htl pathway activity results in reduced debris clearance, which is part of normal CNS development and when defective can lead to abnormal development and neurodegeneration at later stages [34]. When is Htl signaling in glial cells needed for the establishment of their phagocytic ability? Unexpectedly, our data revealed that this pathway is required in embryonic glia at the beginning of their differentiation, and is dispensable at later stages for controlling phagocytosis. Looking carefully at the glial cell network in the control and *htl* knock down embryos, we observed that the length and complexity of glial protrusions were significantly reduced in *htl* knock down embryos, as compared to their control counterparts, forming “holes” in the network. Since we found many more apoptotic cells to be non-engulfed in mutant embryos than in control embryos, we suggest that glial branches form a network that surveys the developing CNS to allow the sensing, recognition and engulfment of apoptotic neurons at different sites in the CNS. We propose that the initial formation of cell branches in developing embryonic glial cells controlled by FGFR/Htl signaling is required for coverage of all the CNS, which is a prerequisite for proper glial phagocytic function at later stages.

FGFR signaling was shown to regulate branch formation in different cells as part of their subcellular morphogenesis, including neurons, tracheal terminal cells in *Drosophila*, vertebrate oligodendrocytes and others [35–39]. FGFR signaling plays a crucial role during early embryonic development of the mesoderm, where cells generate protrusions as part of the transition of epithelial cells into mesenchymal cells [40, 41]. Our study extends this notion to developing glia, revealing a conserved role of the FGFR pathway in branch formation. Taken together, these previously investigated functions of FGFR signaling in various contexts of subcellular morphogenesis suggest that the formation of protrusions requires FGFR-based instruction to the cytoskeleton at very early stages of cell life to determine the specific cell shape needed for a particular function at later periods of life.

The source of FGF ligands can be diverse under different circumstances. In the *Drosophila* larval CNS, Htl signaling is required for the extension and elaboration of astrocyte processes, which is controlled by the release of Pyr and Tsh, likely by neurons [25]. In the *Drosophila* eye imaginal disc, glial differentiation requires a shift from glia–glia interactions mediated by glial Pyr to glia–neuron interactions mediated by neuronal Tsh [21]. Here, we discovered that in the embryonic CNS, Pyr is required in glia for the formation of glial branches. Given that the mutant phenotypes of *pyr* and *htl* knock down embryos were similar, we suggest that Pyr is the main ligand for Htl in the embryonic CNS. However, based on the *tsh* mutant phenotype, we propose that Tsh also plays a role in this process. Our results further demonstrate that generation of the specific multi-branched shape of developing embryonic glia as part of the elaborate glial network relies on autonomous glia-specific Htl signaling. Yet, since *htl*-deficient glial cells still present protrusions and partially perform phagocytosis of apoptotic neurons, we propose that additional cues are involved in glial cell shape formation. However, we have provided strong in vivo evidence here that phagocytic glia require FGFR signaling to affect their subcellular morphogenesis and later become potent phagocytes.

## Methods and experimental procedures

### Fly strains and constructs

All stocks were maintained on standard *Drosophila* media at 25 °C. The following fly strains were used in this work: *repoGal4* (B. Jones), *UAScytGFP* (#1521, Bloomington Stock Center) and *gcmGal4/CyO* (#35541, Bloomington Stock Center), *UASpyrRNAi* (#GD36524, VDRC). The *htl*^*zz81*^*/CyO, pyr*^*s0439*^*/CyO* and *ths*^*759*^*/CyO* mutants, and *UAShtlRNAi*, *UAS-λ-htl (UAShtl-CA)* flies were kindly provided by M. Freeman (Vollum Institute). *UAShtlRNAi* flies (#GD35024) were kindly provided by E. Schejter (Weizmann Institute). *gcmGal4/CyOKrGFP;repoGal4,UAScytGFP/TM6B* was used in a screen when crossed with transgenic lines carrying *UASRNAi* for candidate genes. Information about candidate genes tested in the screen is provided in Supplementary Fig. 1. The *w1118* strain served as a wild-type control.

### Immunohistochemistry and imaging

For immunohistochemistry, embryos were fixed and stained according to standard procedures. Guinea pig anti-SIMU antibodies [42] was used at a 1:5000 dilution, while rabbit anti-Htl antibodies, a gift from T. Kojima, were used at a 1:1000 dilution. Rabbit anti-activated Dcp-1 (Cell signaling) and mouse anti-GFP (Roche) antibodies were used at a 1:100 dilution each. Mouse anti-REPO, mouse anti-Eve and mouse anti-CUT (Developmental Studies Hybridoma Bank) antibodies were used at 1:20, 1:100 and 1:5 dilutions, respectively. Fluorescent secondary antibodies (Cy3- and Cy5-labeled antibodies from Jackson ImmunoResearch and Alexa Fluor 488-labeled antibodies from Molecular Probes) were used at 1:200 dilutions each. A 75% glycerol solution was used as imaging medium. Microscope imaging and data analyses of all images were acquired on a Zeiss AxioImager M2 microscope equipped with an ApoTome2 optical sectioning device. All images taken using Zen acquisition software and processed with Adobe Photoshop CS6.

### Evaluation of glial branches

To measure number and lengths of glial branches, we labeled SIMU-marked glial extensions using Imaris software and analyzed glial cells located in the central part of five abdominal segments in each embryo. Branches that extended from the midline (not in parallel and labeled in yellow in Fig. 6a’, b’) were counted, including the number of bifurcations, their lengths and surface areas (Supplementary Movies 3 and 4), using Imaris (Bitplane) software. Five embryos of each genotype were analyzed, assessing five abdominal segments in each embryo. To evaluate the inside/outside position of apoptotic particles relative to phagocytic glia, rotations of Z-stacks were performed using Imaris (Bitplane) software, as shown in Supplementary Movies 1 and 2 and Supplementary Fig. S2. Imaris analysis was performed blinded on acquired apotome images.

### Quantitative and statistical analysis

Embryos of each genotype [5–9] were tested (*n* = number of embryos, indicated in each figure legend). All quantifications were performed using Imaris (Bitplane) software with an appropriate iso-surfacing threshold. Volume of apoptotic particles (labeled with anti-Dcp-1 antibodies) and number of Eve-positive neurons were measured in 15 Z-stacks (0.52 µm each, total 7.8 µm) in cortex area of the embryonic CNS. The numbers of REPO-positive nuclei and Cut-positive cells were assessed in 6 and 10 Z-stacks, respectively (0.52 µm each, total 3.12 µm or 5.2 µm), in the cortex area of the embryonic CNS. Quantification of branches, their surface areas and the positions of apoptotic particles inside/outside glial cells was performed in 6 Z-stacks (0/52 µm each, total 3.12 µm).

To determine statistical significance, Prism GraphPad, version 8, software was employed. First, Normality and Log normality were assessed using a Shapiro–Wilk test. In some experiments, there was no normal distribution, and, therefore, non-parametric tests were conducted. An average of all experiments is shown as the mean and SD ± 95% confidence interval and the number (*n*) of embryos examined. *p* values were generated using two-tailed Mann–Whitney or Kruskal–Wallis tests (depending on the number of samples) to compare different genotypes.

## Supplementary information

Supplemental Material

Supplementary movie 1

Supplementary movie 2

Supplementary movie 3

Supplementary movie 4

## Data Availability

All data generated or analyzed during this study are included in the main text or the Supplementary Materials.
