# Supplementary material for: Drosophila FGFR/Htl signaling shapes embryonic glia to phagocytose apoptotic neurons
Source: Cell Death Discov. 2023 Mar 10;9:90. doi: 10.1038/s41420-023-01382-5 (PMC10006210; doi:10.1038/s41420-023-01382-5)
Supplement: Supplementary file 1 — Supplemental Material [file 41420_2023_1382_MOESM1_ESM.docx]

**Supplementary material**

**
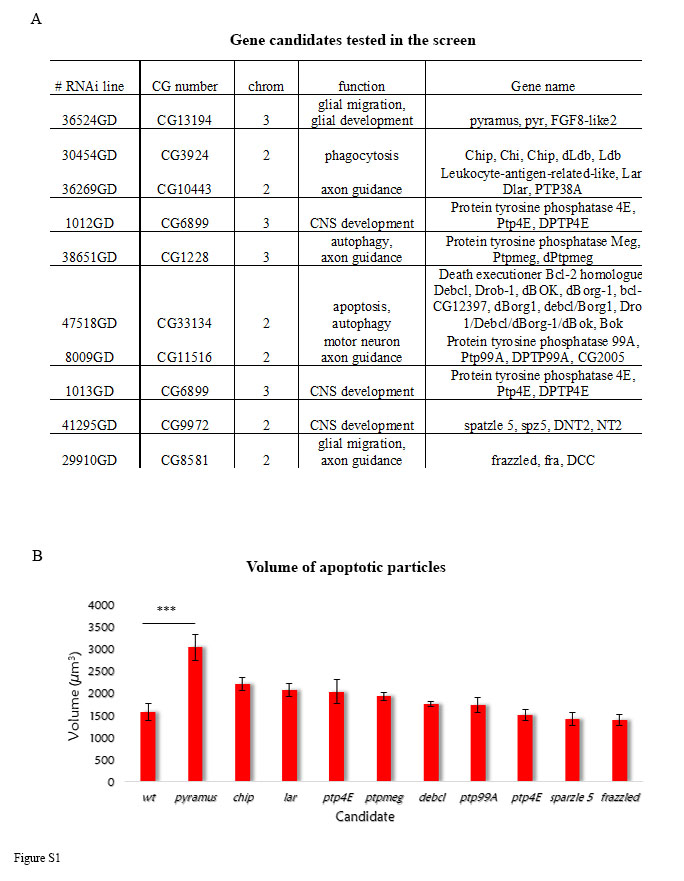
**

**Supplementary Figure 1. Candidate screen for genes involved in glial phagocytosis of apoptotic neurons during embryogenesis.** (A) Table of gene candidates, including the number of RNAi line, CG number, chromosome location, known function and names, based on Flybase data. (B) Mean total volume of apoptotic particles within CNS sections ± SEM, n=8. Statistical significance was analyzed employing one-way ANOVA, ***p<0.001.

**
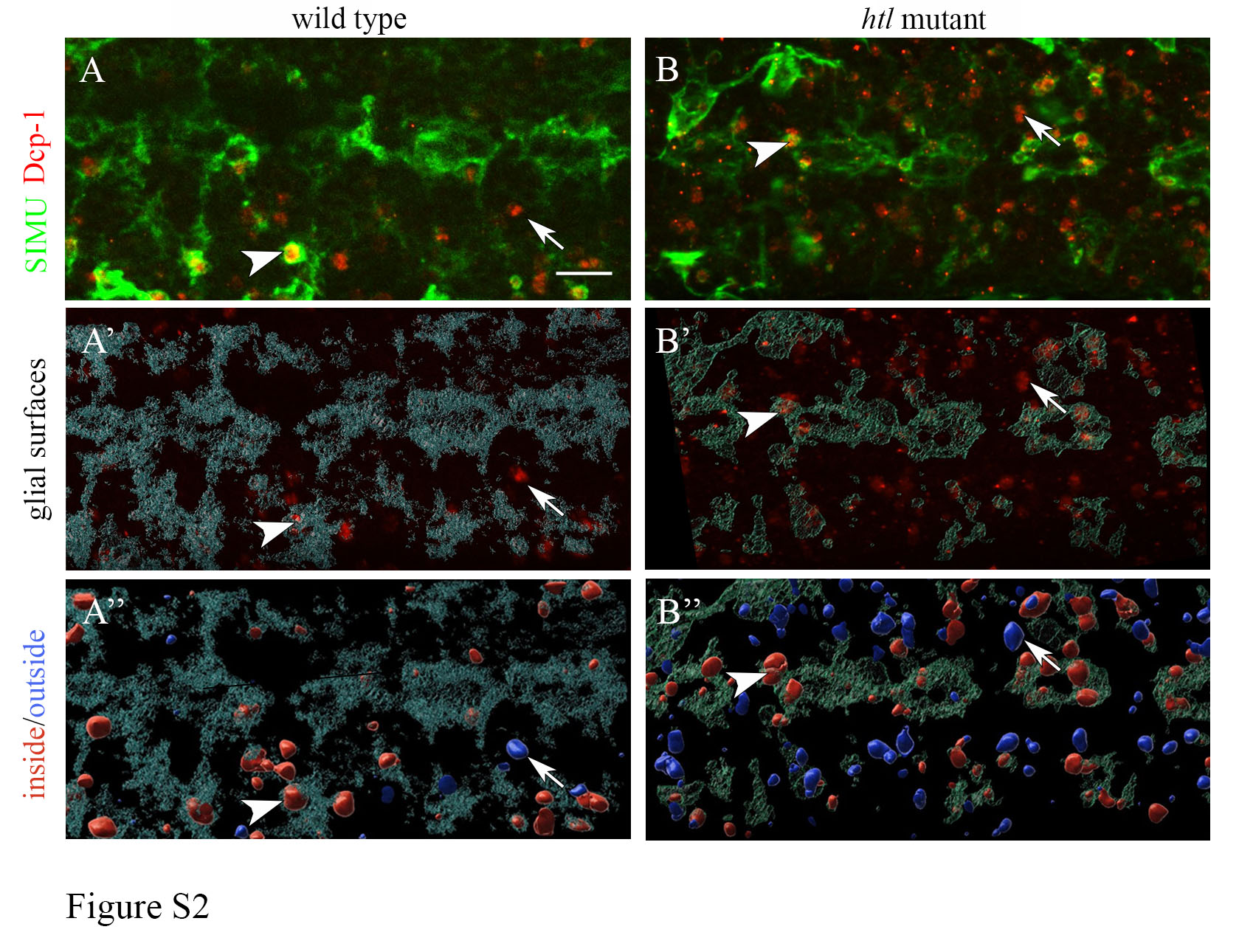
**

**Supplementary Figure 2 (stills taken from movies 1 and 2). Evaluation of localization of apoptotic particles inside/outside glial cells.** (A-B'') 3D projections of the embryonic CNS at stage 16; ventral view. Bar, 20 µm. (A-A'') Wild type and (B-B'') *htl^zz81^* heterozygous embryos. (A,B) Glial cell membranes are labeled with anti-SIMU antibodies (green) and apoptotic particles were labeled with anti-Dcp-1 antibodies (red). (A',B') The Imaris Surfaces tool was applied to the A and B images. (A'',B'') An additional Imaris Surfaces tool was applied to apoptotic particles. Arrows depict apoptotic particles outside glial cells, while arrowheads depict apoptotic particles inside glia.

**Movies 1,2: Localization of apoptotic particles inside/outside glial cells. (**1) Wild type embryo; (2) *htl^zz81^* heterozygous embryo. Glial cell membranes were labeled with anti-SIMU antibodies (green), while apoptotic particles with anti-Dcp-1 antibodies (red). Glial surfaces were marked with the Imaris Surfaces tool. By rotating the 3D stack of the imaged area of the embryonic CNS, we were able to distinguish between inside particles (marked in strong red) and outside particles (marked in blue). Stills from these movies are presented in Supplementary Figure 2.

**Movies 3,4: Evaluation of glial surface area.** (3) Control embryo (*gcmGal4;repoGal4,cytGFP)*; (4) mutant embryo (*gcmGal4;repoGal4,htlRNAi)*. Glial cell membranes were labeled with anti-SIMU antibodies (green). Glial surfaces were marked with the Imaris Surfaces tool. The total surface area of marked glial cells was extracted from the Imaris data.
